# Targeting PIM kinase as a therapeutic strategy in human hepatoblastoma

**DOI:** 10.18632/oncotarget.25205

**Published:** 2018-04-27

**Authors:** Laura L. Stafman, Smitha Mruthyunjayappa, Alicia M. Waters, Evan F. Garner, Jamie M. Aye, Jerry E. Stewart, Karina J. Yoon, Kimberly Whelan, Elizabeth Mroczek-Musulman, Elizabeth A. Beierle

**Affiliations:** ^1^ Department of Surgery, University of Alabama at Birmingham, Birmingham, AL, USA; ^2^ Department of Pathology, University of Alabama at Birmingham, Birmingham, AL, USA; ^3^ Department of Pediatrics, University of Alabama at Birmingham, Birmingham, AL, USA; ^4^ Department of Pharmacology and Toxicology, University of Alabama at Birmingham, Birmingham, AL, USA

**Keywords:** hepatoblastoma, PIM kinase, AZD1208, HuH6

## Abstract

Increasing incidence coupled with poor prognosis and treatments that are virtually unchanged over the past 20 years have made the need for the development of novel therapeutics for hepatoblastoma imperative. PIM kinases have been implicated as drivers of tumorigenesis in multiple cancers, including hepatocellular carcinoma. We hypothesized that PIM kinases, specifically PIM3, would play a role in hepatoblastoma tumorigenesis and that PIM kinase inhibition would affect hepatoblastoma *in vitro* and *in vivo*. Parameters including cell survival, proliferation, motility, and apoptosis were assessed in human hepatoblastoma cells following PIM3 knockdown with siRNA or treatment with the PIM inhibitor AZD1208. An *in vivo* model of human hepatoblastoma was utilized to study the effects of PIM inhibition alone and in combination with cisplatin. PIM kinases were found to be present in the human hepatoblastoma cell line, HuH6, and in a human hepatoblastoma patient-derived xenograft, COA67. PIM3 knockdown or inhibition with AZD1208 decreased cell survival, attachment independent growth, and motility. Additionally, inhibition of tumor growth was observed in a hepatoblastoma xenograft model in mice treated with AZD1208. Combination therapy with AZD1208 and cisplatin resulted in a significant increase in animal survival when compared to either treatment alone. The current studies showed that PIM kinase inhibition decreased human hepatoblastoma tumorigenicity both *in vitro* and *in vivo*, implying that PIM inhibitors may be useful as a novel therapeutic for children with hepatoblastoma.

## INTRODUCTION

Hepatoblastoma is the most common primary liver malignancy in the pediatric population with an incidence of 4 per million in children younger than 5 years [1], the age when the majority of patients are diagnosed. Prognosis worsens with increasing age at diagnosis such that those younger than 5 years have a 5-year survival of 64% compared to only 20% for those 15-19 years old [2]. The incidence of hepatoblastoma has increased 4.3% from 1992 to 2004, yet treatment has not changed significantly in the past 20 years [3]. Given the increased incidence coupled with poor prognosis, novel therapies must be developed for these children.

Proviral Integration site for Maloney murine leukemia virus kinases, or PIM kinases (PIM1, PIM2, PIM3) are a family of serine/threonine kinases that promote tumorigenesis in a variety of cancers including leukemia, lymphoma, prostate, gastric, colorectal and hepatocellular carcinoma [4–10] through downstream proteins associated with the cell cycle [11–14], migration [8], and apoptosis [15]. Many of the substrates of PIM kinases are still being determined, but some of the well-documented target proteins include the pro-apoptotic protein, BAD [15], and the cell cycle inhibitor, p21 [13]. To our knowledge, PIM kinases have not previously been examined in hepatoblastoma. Other investigators have found that PIM3, specifically, plays a role in the development of hepatocellular carcinoma [9, 16], a liver cancer that occurs predominantly in adults. These investigators demonstrated that transgenic mice selectively expressing PIM3 in the liver developed hepatocellular carcinoma more frequently than wild type mice, and that inhibition of PIM3 decreased cell proliferation and apoptosis in hepatocellular carcinoma [9, 16]. These data led us to hypothesize that PIM3 may play a role in hepatoblastoma tumorigenesis and that PIM kinase inhibition would affect hepatoblastoma tumor growth *in vitro* and *in vivo*. Examination of PIM3 kinase in hepatoblastoma has not yet been recorded in the literature, making this a unique study and a potential novel therapeutic approach to hepatoblastoma.

## RESULTS

### PIM kinases were expressed in HuH6 cells, COA67 patient-derived xenograft (PDX) cells, and human hepatoblastoma tumor specimens

**Figure 1:**
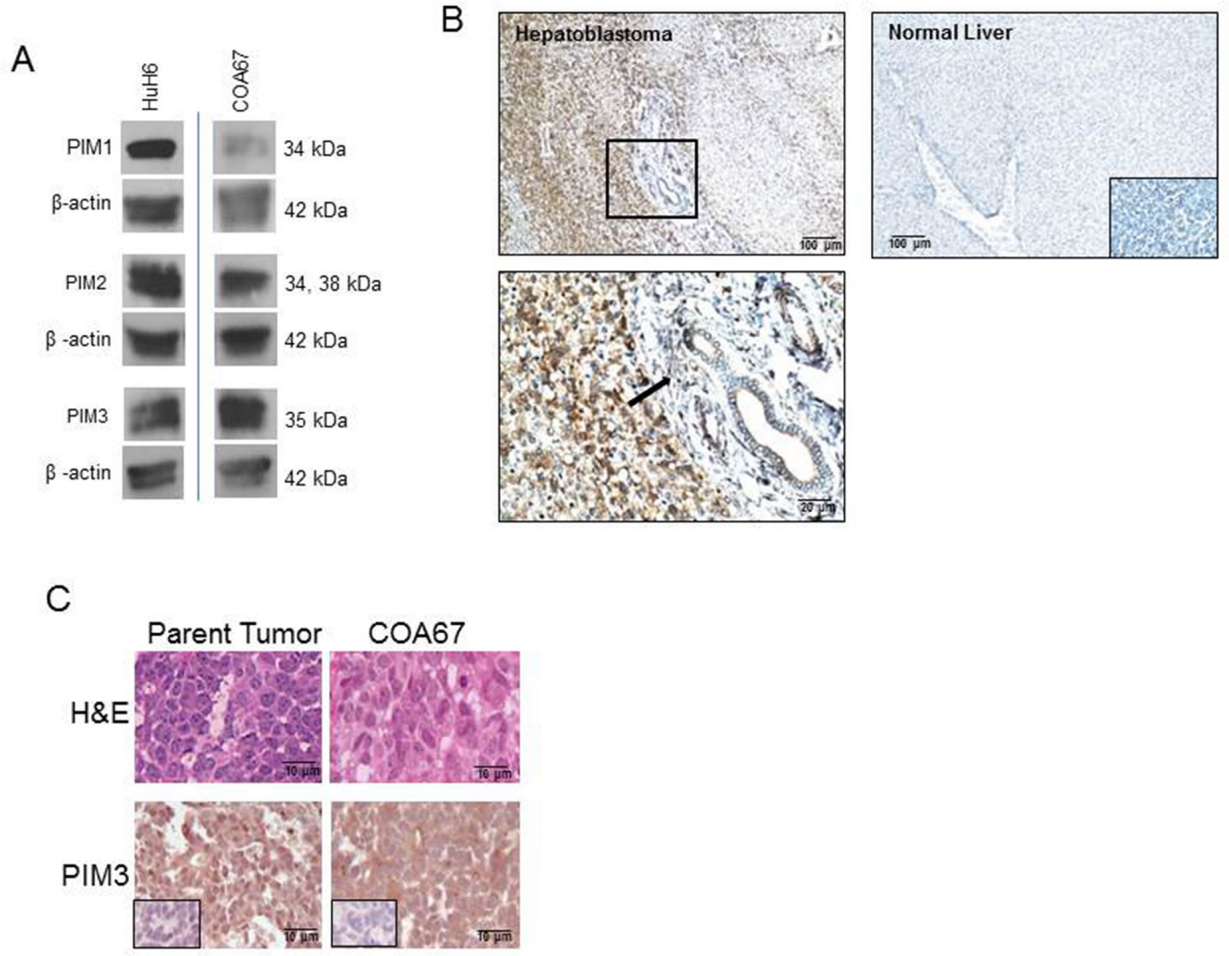
PIM kinases were expressed in HuH6 cells, the COA67 PDX, the parent tumor for COA67, and human hepatoblastoma tumor specimens **(A)** Immunoblotting for PIM1, PIM2, and PIM3 was completed on HuH6 and COA67 cell lysates with β-actin controls. All three PIM proteins were present in both cell lines. **(B)** Immunohistochemistry for PIM3 was performed on human hepatoblastoma (*top left panel, magnification on bottom left panel*) and normal liver (*top right panel*) with negative staining in the IgG control is in bottom right insert, not bottom right panel as currently stated. PIM3 was more highly expressed in hepatoblastoma (*top left panel*) than normal liver (*top right panel*). Black arrow in bottom left panel indicates lack of staining in the peri-portal connective tissue in comparison to the PIM3 stained hepatoblastoma cells in the left half of the photomicrograph. **(C)** Hematoxylin and eosin (*top panel*) and immunohistochemistry for PIM3 (*bottom panel*) with IgG control (*inserts, bottom panels*) in parent tumor (*left bottom panel*) and COA67 PDX (*right bottom panel*) revealed that the COA67 PDX closely resembled the parent tumor histomorphologically and PIM3 was present in both the PDX and its parent.

The expression of PIM kinases (PIM1, PIM2, and PIM3) was evaluated in the long-term passaged human hepatoblastoma cell line, HuH6, and the human hepatoblastoma PDX cell line, COA67, by immunoblotting. Western blotting demonstrated the presence of PIM1, PIM2, and PIM3 (Figure 1A). PIM3 expression was found to be present in human hepatoblastoma specimens by immunohistochemistry and less expression was observed in normal human liver tissue. Representative photomicrographs are presented in Figure 1B. Rabbit IgG was used as a control and reacted appropriately. The enlarged image reveals the PIM3 staining of hepatoblastoma cells in contrast with the lack of staining in the peri-portal connective tissue of the liver (Figure 1B, *lower left panel, black arrow*).

Hematoxylin and eosin (H&E) staining of the parent hepatoblastoma tumor and the COA67 PDX confirmed that the PDX closely resembles the original tumor, consisting mainly of small round blue cells and scant stroma (Figure 1C, *upper panels*). Immunohistochemical (IHC) staining for PIM3 in the parent and COA67 PDX tumor showed that PIM3 was present in both the human parent tumor from which COA67 was derived and a COA67 PDX tumor specimen harvested from a mouse (Figure 1C, *lower panels*). IgG negative controls stained appropriately (Figure 1C, *inserts, lower panels*).

### PIM3 kinase inhibition with siRNA decreased proliferation and migration in HuH6 hepatoblastoma cells

**Figure 2:**
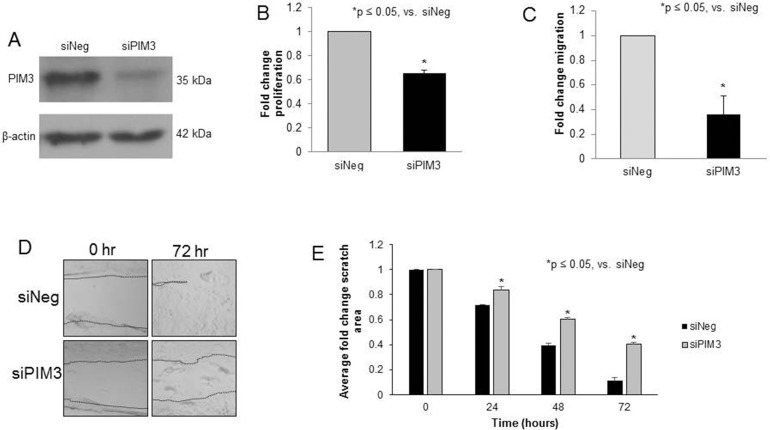
PIM3 kinase inhibition with siRNA decreased proliferation and migration in HuH6 hepatoblastoma cells **(A)** HuH6 cells were transfected with siRNA targeting PIM3 (siPIM3) or non-targeting control (siNeg) for 5 days, after which the following experiments were performed. Immunoblotting confirmed knockdown of PIM3 in the siPIM3 cells compared to the siNeg treated cells. **(B)** Using the CellTiter 96® assay to assess proliferation, the siPIM3 cells had significantly decreased fold change in proliferation compared to the siNeg cells. **(C)** Transfected cells were allowed to migrate through the 8μm pore membrane of Transwell plates. After 24 hours, cells were fixed, stained, and counted. siPIM3 transfected cells exhibited significantly decreased migration compared to siNeg cells. **(D)** HuH6 cells were plated and allowed to reach 80% confluence. A 200 μL pipette tip was utilized to create a standard scratch on the plate and the plates were examined every 24 hours up to 72 hours. Representative photographs of plates at 72 hours show decreased motility across the scratch in siPIM3 cells compared to siNeg cells. Location of scratch borders are emphasized by a dotted line. **(E)** Area of the scratch remaining was quantified in pixels using ImageJ software with data reported as fold change in scratch area ± SEM. siPIM3 transfected cells exhibited significantly less motility across the scratch than siNeg transfected cells at all time points examined.

SiRNA was utilized to target PIM3 (siPIM3). Western blotting confirmed that pooled siPIM3 decreased expression of PIM3 in HuH6 cells (Figure 2A). Data regarding individual pool members’ effect on PIM3 expression and proliferation is included as Supplementary Materials (Supplementary Figure 1). Proliferation decreased 34% in HuH6 cells transfected with siPIM3 compared to siNeg controls (p = 0.01, Figure 2B). When examining migration, HuH6 cells transfected with siPIM3 exhibited 63% less migration through Transwell inserts compared to siNeg controls (p < 0.05, Figure 2C). These results were confirmed with a cell monolayer wounding assay in which HuH6 cells transfected with siPIM3 crossed the wounded monolayer significantly less than siNeg cells (Figure 2D, 2E). On average, 41% of the scratch remained free of cells on the siPIM3 plates at 72 hours compared to only 11% on the siNeg plates. siRNA targeting the other members of the PIM family, PIM1 and PIM2, in HuH6 cells revealed a decrease in protein expression with no significant change in cell proliferation (Supplementary Figure 2).

### PIM kinase inhibition with AZD1208 decreased proliferation, motility, and attachment-independent growth in HuH6 hepatoblastoma cells

**Figure 3:**
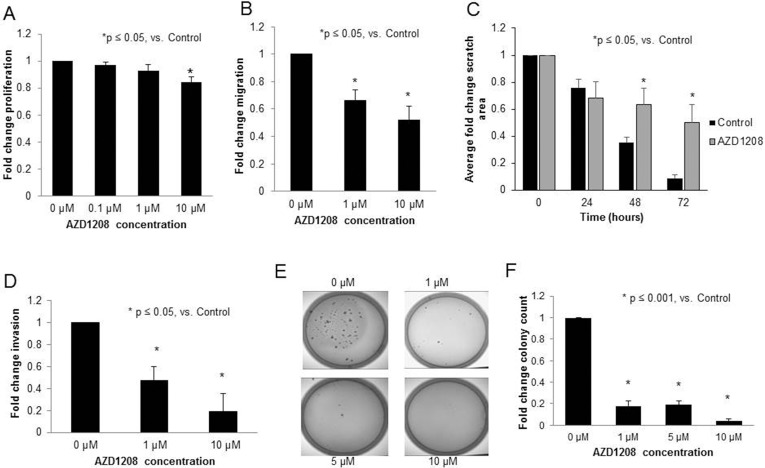
PIM kinase inhibition with AZD1208 decreased proliferation, migration, invasion, and attachment-independent growth in HuH6 hepatoblastoma cells **(A)** Following 24 hours of treatment with 10 μM AZD1208, the proliferation of HuH6 cells measured with CellTiter 96® assay was significantly decreased compared to the control. **(B)** HuH6 cells treated with increasing doses of AZD1208 were allowed to migrate for 24 hours then fixed, stained, and counted. HuH6 cells treated with AZD1208 exhibited significantly decreased migration compared to untreated cells. **(C)** HuH6 cells were plated and allowed to reach 80% confluence. The media was changed for fresh untreated or treated (10 μM AZD1208) media and a standard scratch was placed on the plate using a 200 μL pipette tip. Scratches were imaged every 24 hours up to 72 hours. Area of the scratch remaining was quantified in pixels using ImageJ software with data reported as fold change in scratch area ± SEM. **(D)** For invasion, AZD1208 treated cells were allowed to invade for 24 hours, then fixed, stained, and counted. Cells treated with AZD1208 had significantly decreased invasion compared to untreated cells. **(E)** Soft agar assays were used to assess attachment-independent growth. HuH6 cells were treated with increasing concentrations of AZD1208, grown in soft agar for 1 month, and colonies were imaged and counted. Representative photographs of plates show decreased numbers of colonies in AZD1208 treated versus control plates. **(F)** Soft agar colonies were quantified with ImageJ. Colony count was significantly decreased with AZD1208 treated compared to untreated cells. All experiments were repeated at least in triplicate and data reported as fold change ± SEM.

For the remainder of the experiments, AZD1208 was used to achieve PIM inhibition in anticipation of advancing to *in vivo* studies. The effects of PIM inhibition with AZD1208 was evaluated using a number of methods. First, proliferation was examined. AZD1208 treatment resulted in a 16% decrease in proliferation at a concentration of 10 μM in the HuH6 cell line (p<0.05, Figure 3A). Since tumor metastasis is a hallmark of aggressive hepatoblastoma, cell migration, invasion, and attachment independent growth were next evaluated. Following treatment with AZD1208, migration of HuH6 cells was significantly decreased as seen by Transwell plate and monolayer wounding assay (Figure 3B, 3C, respectively). AZD1208 treatment also significantly decreased HuH6 cell invasion (Figure 3D). Attachment independent growth was significantly inhibited after treatment with AZD1208 (Figure 3E, 3F).

### PIM kinase inhibition with AZD1208 induced cell cycle arrest and apoptosis in HuH6 hepatoblastoma cells

**Figure 4:**
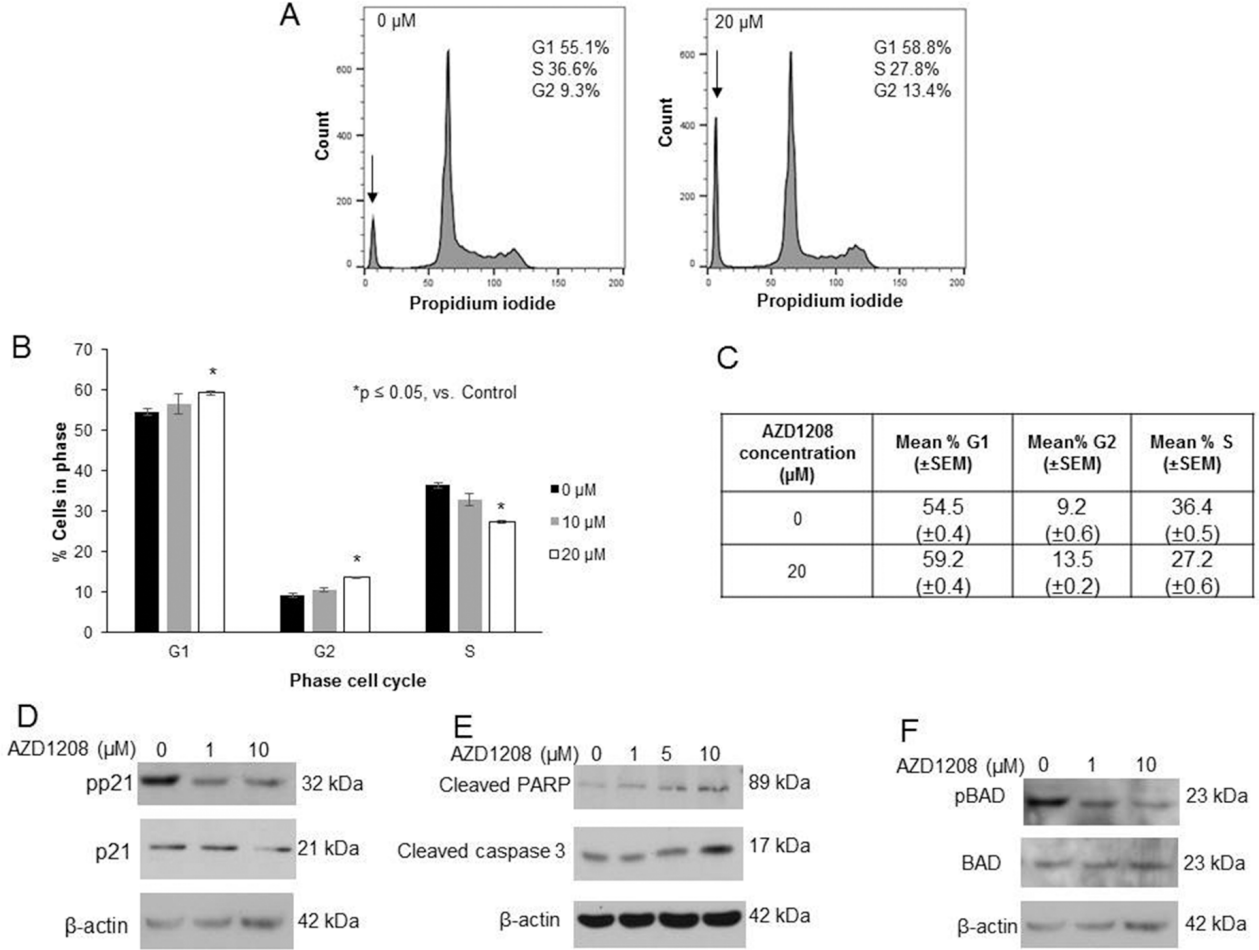
AZD1208 prevented progression through the cell cycle and led to apoptosis in HuH6 hepatoblastoma cells **(A)** Cells were treated with 0, 10 or 20 μM AZD1208 for 72 hours and cell cycle was analyzed with propidium iodide staining using flow cytometry. Representative histograms showing relative percentage of cells in each phase of the cell cycle show that there was a decrease in S phase and increase in G_1_ and G_2_ phases with PIM inhibition. There was also an increase in the sub-G_1_ population (indicated by the arrow, 23.4% versus 12.7% of the total histogram) in cells treated with AZD1208 versus control. **(B)** Quantification of average percent cells in each phase of the cell cycle across all replicates revealed a significant decrease in S phase, and increase in G_1_ and G_2_ phases in cells treated with 20 μM AZD1208 compared to control. **(C)** Tabular representation of the average percent cells in each phase of the cell cycle across all replicates revealing a significant decrease in S phase, increase in G_1_ phase, and increase in G_2_ phase in cells treated with 20 μM AZD1208 compared to control. Data represent the mean ± SEM. **(D)** Immunoblotting for phospho-p21 (T145) and total p21 in HuH6 cells showed a decrease in p21 phosphorylation with 24 hour AZD1208 treatment with no accompanying change in total p21 protein. **(E)** Immunoblotting for cleaved PARP and cleaved caspase 3, markers of apoptosis, in HuH6 cells revealed an increase in cleaved products in both markers with increasing concentrations of AZD1208 at 24 hours. **(F)** Immunoblotting for phospho-BAD (S112) in HuH6 cells showed that BAD phosphorylation was decreased with AZD1208 at 24 hours whereas there was no change in total BAD; evidence for apoptosis.

To further examine the phenotypic changes observed with PIM kinase inhibition, cell cycle progression was analyzed. AZD1208 resulted in an arrest of cell cycle progression in HuH6 cells, indicated by an increased percentage of cells in the G_1_ and G_2_ phases accompanied by a decreased percentage of cells in the S phase (Figure 4A-4C). Representative histograms are presented in Figure 4A. PIM kinases have been demonstrated to phosphorylate the Thr145 site of cyclin dependent kinase inhibitor p21, resulting in cytoplasmic localization of p21, where it is unable to perform its normal function to arrest the cell cycle [13]. Because AZD1208 inhibited progression through the cell cycle in HuH6 cells, we sought to determine whether p21 was affected by PIM inhibition in these cells. AZD1208 treatment in HuH6 cells led to a decrease in phosphorylation of p21 at the Thr145 site without changing expression of total p21 (Figure 4D), providing further evidence of AZD1208-induced cell cycle arrest.

In addition to changes in cell cycle progression, we examined whether PIM inhibition in HuH6 cells resulted in apoptosis. We observed an increase in the sub-G_1_ population on cell cycle analysis with AZD1208 treatment, which is consistent with apoptosis. The sub-G_1_ population, indicated by the arrow in Figure 4A, accounted for 23.4% (±0.1%) of the cell cycle histograms for cells treated with 20 μM AZD1208 but only 13.7% (±0.8%) of the histograms for untreated cells. Additionally, the presence of apoptosis was indicated by an increase in cleaved PARP and cleaved caspase 3 by immunoblotting (Figure 4E). PIM kinases have been shown to directly phosphorylate the pro-apoptotic protein BAD at the Ser112 site [15, 17]. In this study, PIM inhibition with AZD1208 decreased phosphorylation of BAD (Figure 4F), which is another indicator of apoptosis.

### PIM kinase inhibition decreased *in vivo* tumor growth in a mouse model of hepatoblastoma

**Figure 5:**
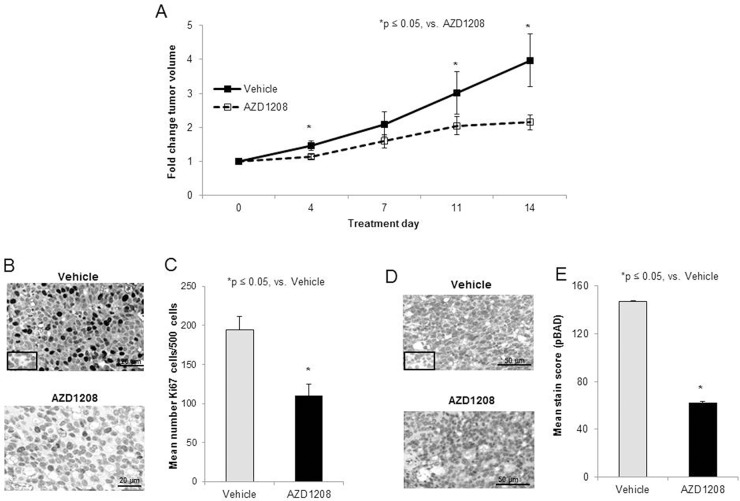
PIM kinase inhibition decreased tumor growth in a xenograft model of hepatoblastoma **(A)** Human hepatoblastoma cells, HuH6 (2.5 x 10^6^ cells), were injected into the right flank of female athymic nude mice. When tumor sized reached an average of 150 mm^3^, mice were randomized to receive vehicle or AZD1208 by oral gavage for a total of 14 days (n = 15 per group). Tumor growth was followed and tumors were measured twice weekly with calipers to calculate tumor volumes. Mice treated with AZD1208 had significantly smaller tumors compared with vehicle-treated mice, reported as fold change tumor volume ± SEM. **(B)** Formalin-fixed, paraffin-embedded tumor xenografts were stained for Ki67, a marker of cell proliferation, and representative photomicrographs of vehicle-treated (*top panel*) and AZD1208-treated (*bottom panel*) xenografts are shown with IgG control in the bottom left corner (*top panel*). **(C)** The number of Ki67 cells per 500 cells in a representative section of each tumor was counted and the mean was calculated and reported for each group ± SEM. AZD1208-treated xenografts had significantly decreased Ki67 staining compared to vehicle-treated xenografts. **(D)** Formalin-fixed, paraffin-embedded tumor xenografts were stained for phospho-BAD (S112). When BAD is dephosphorylated, apoptosis is believed to occur. Representative photomicrographs of vehicle-treated (*top panel*) and AZD1208-treated (*bottom panel*) xenografts are shown with IgG control in the bottom left corner (*top panel*). **(E)** Slides stained for phospho-BAD were scored based upon the intensity of the stain and the percentage of tumor cells staining positive. AZD1208-treated xenografts had significantly decreased phospho-BAD staining compared to vehicle-treated xenografts, indicated an increase in apoptosis with AZD1208 treatment, reported as mean stain score ± SEM.

Studies were then advanced to an *in vivo* flank model of hepatoblastoma. After 2 weeks of treatment, mice treated with AZD1208 had significantly smaller tumors than control mice (Figure 5A). At the time of euthanasia, the average tumor size in the control mice was 346.3 ± 54.3 mm^3^ compared to 225 ± 42.3 mm^3^ in the AZD1208-treated mice (p < 0.05 AZD1208 vs. control). The animals’ weights were not significantly affected by the treatment (Supplementary Figure 3). The tumors were examined for cell proliferation using immunohistochemical staining for Ki67. Representative photomicrographs demonstrate decreased Ki67 staining in AZD1208 treated tumors compared to control tumors (Figure 5B, *upper and lower panels*), indicating decreased proliferation in the AZD1208 treated tumors. When quantified, there was significantly less Ki67 staining in the treated tumors (Figure 5C). Since BAD is a known downstream target of PIM kinase [15, 17], and PIM kinase inhibition decreased BAD phosphorylation in HuH6 cells *in vitro* (Figure 4E), sections of the HuH6 tumors were stained for phosphorylated BAD. Representative photomicrographs demonstrate less staining for phospho-BAD in tumors from AZD1208-treated mice versus vehicle-treated control mice (Figure 5D, *upper and lower panels*). When quantified, there was a significant decrease in phospho-BAD staining in the AZD1208-treated group compared to the control vehicle-treated group (Figure 5E).

**Figure 6:**
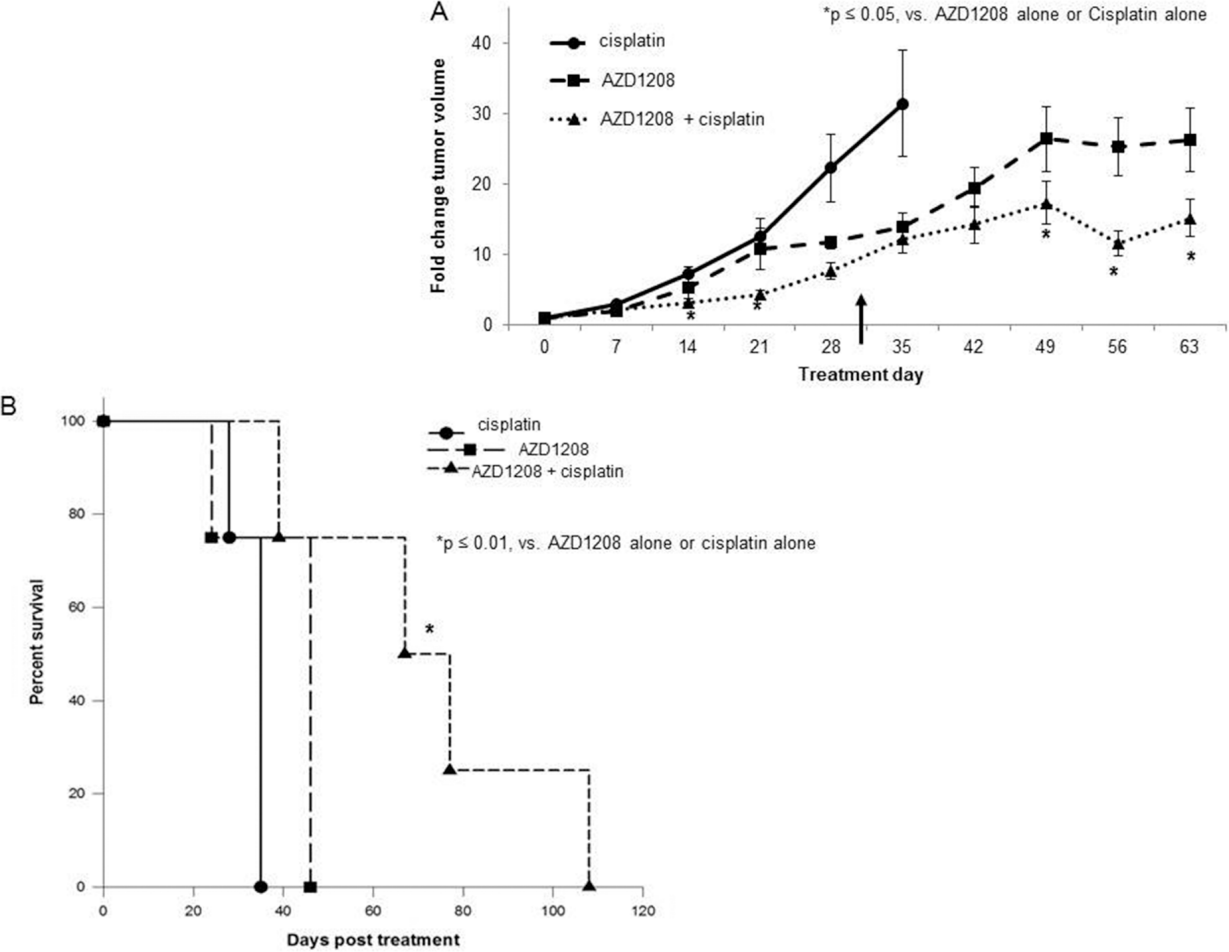
PIM inhibition in combination with cisplatin decreased tumor growth and increased survival in a xenograft model of hepatoblastoma HuH6 cells (2.5 x 10^6^ cells) were injected subcutaneously into the right flank of female athymic nude mice. When tumor size reached greater than 45 mm^3^ for all animals included in the study, mice were randomized to receive cisplatin alone, AZD1208 alone, or combination treatment with cisplatin and AZD1208 administered in the same doses as in the single agent groups (n = 4 per group). Mice received cisplatin (or sterile saline for AZD1208 alone group) by intraperitoneal injection on days 1-3 and 14-16. On the remaining days (days 4-13 and 17-30), mice received AZD1208 in ORA-Plus® (or ORA-Plus® alone for cisplatin alone group) by oral gavage. Tumor growth was followed and tumors were measured twice weekly with calipers to calculate tumor volumes, which were reported as fold change tumor volume ± SEM. **(A)** Mice treated with the combination of AZD1208 and cisplatin had significantly smaller tumors compared with either monotherapy-treated mice. The upward pointing black arrow indicates the last day of treatment received. **(B)** Mice with were followed for survival and humanely euthanized when IACUC parameters were met. Animal survival was compared with log-rank test. Animals treated with the combination of AZD1208 and cisplatin had significantly improved survival compared with those treated with AZD1208 or cisplatin alone.

Current therapies for hepatoblastoma are based upon cisplatin [18], so we next examined the effect of combining AZD1208 with cisplatin therapy. HuH6 cells were injected into the right flank of female athymic nude mice (N=12). Once tumors reached 45 mm^3^, animals were randomized to receive cisplatin alone (2 mg/kg body weight/day), AZD1208 alone (15 mg/kg body weight/day), or combination treatment with cisplatin and AZD1208 in the same doses as in the single agent for 30 days (treatment schema Supplementary Figure 4). Animals were followed for survival. Tumors in those mice receiving monotherapy grew more rapidly than those receiving the combination of AZD1208 and cisplatin (p < 0.05, Figure 6A). Mean survival following initiation of treatment was 33.2 ± 1.8 days in mice treated with cisplatin alone, 40.5 ± 5.5 days in those treated with AZD1208 alone, and 72.8 ± 14.2 days in those treated with combination therapy (Figure 6B). There was no significant difference in survival between the groups receiving AZD1208 alone or cisplatin alone (p = 0.14), but there was a significant difference between monotherapy and combination therapy (p = 0.01 for cisplatin alone vs. combination therapy, p = 0.01 for AZD1208 alone vs. combination therapy). The animals’ weights were not significantly affected by the treatments (Supplementary Figure 5).

### PIM kinase inhibition with AZD1208 decreased survival, proliferation, migration, and invasion, and induced apoptosis in the hepatoblastoma PDX COA67

**Figure 7:**
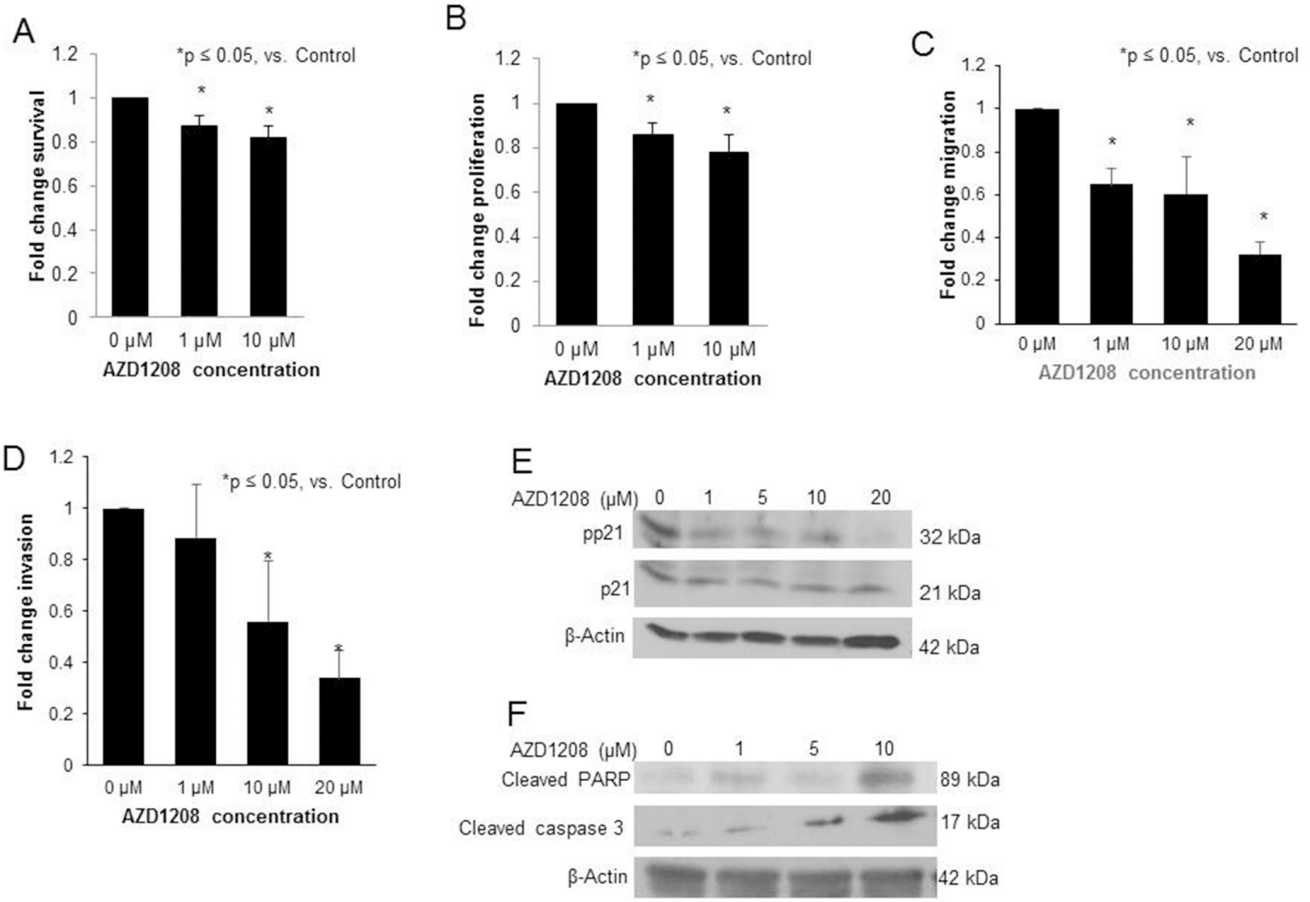
PIM kinase inhibition with AZD1208 decreased survival, proliferation, migration, and invasion, affected phosphorylation of p21, and induced apoptosis in the hepatoblastoma PDX, COA67 **(A)** Following 24 hours of treatment with AZD1208, the survival of COA67 cells was assessed with the alamarBlue® Assay. Cells treated with AZD1208 had significantly decreased viability compared to the control cells. **(B)** Following 24 hours of treatment with AZD1208, the proliferation of COA67 cells was assessed. Cells treated with AZD1208 had significantly decreased proliferation compared to the control cells, reported as fold change ± SEM. **(C)** COA67 cells treated with increasing doses of AZD1208 were allowed to migrate through a micropore membrane for 72 hours. Migration was reported as fold change. COA67 cells treated with AZD1208 exhibited significantly decreased migration compared to untreated cells. **(D)** For invasion, AZD1208-treated cells were allowed to invade for 72 hours. Cells treated with AZD1208 exhibited significantly decreased invasion compared to untreated cells. All experiments were repeated at least in triplicate, with results reported as fold change ± SEM. **(E)** Immunoblotting for phospho-p21 (T145) and total p21 in COA67 cells showed a decrease in phospho-p21 with AZD1208 treatment with no accompanying change in total p21. **(F)** Immunoblotting for cleaved PARP and cleaved caspase 3, markers of apoptosis, in COA67 cells revealed an increase in both markers with increasing concentrations of AZD1208.

Since patient derived xenografts (PDXs) may more closely represent the human condition [19], PIM3 inhibition was evaluated using a hepatoblastoma PDX (COA67). AZD1208 treatment resulted in a significant decrease in COA67 cell survival and cell proliferation at a concentration of 1 μM (Figure 7A, 7B, respectively). Following treatment with AZD1208, migration and invasion of COA67 cells were both decreased significantly (Figure 7C, 7D, respectively). AZD1208 decreased phosphorylation of the cell cycle regulator, cyclin dependent kinase inhibitor p21, at the Thr145 site in COA67 PDX hepatoblastoma cells with no change in total p21 expression (Figure 7E). Given the decrease in cell viability with PIM inhibition, we also sought to examine whether AZD1208 induced apoptosis in COA67 cells. There was an increase in apoptosis with PIM inhibition, demonstrated by an increase in cleaved PARP and cleaved caspase 3 expression (Figure 7F). Therefore, PIM inhibition in a human hepatoblastoma PDX resulted in significant changes in the tumorigenic phenotype, similar to those seen with the long-term passaged hepatoblastoma cell line, HuH6.

## DISCUSSION

PIM kinases have been shown to be expressed in a number of human solid tumors and leukemia [4–10, 20]. In this study, we found that PIM3 kinase was expressed in hepatoblastoma. IHC revealed that PIM3 expression was mostly localized to the cytoplasm of the cells, similar to the finding of other researchers [20]. Further, the expression of PIM3 by IHC in normal liver parenchyma was nearly undetected, leading us to hypothesize that PIM3 inhibition will be cytotoxic to hepatoblastoma cells but not normal liver cells. There are a number of studies that would support this belief. Fujii *et al.* found that PIM3 was not expressed in normal hepatocytes, but was aberrantly expressed in pre-malignant and hepatocellular carcinoma tissues [16]. Additionally, PIM3 knockout mice were viable and fertile without any gross abnormalities [21]. Finally, clinical studies examining the effects of PIM kinase inhibition have not yielded dose limiting toxicities related to hepatic function [22].

PIM3 was specifically targeted with siRNA and resulted in decreased proliferation and cell migration, substantiating the importance of PIM3 in hepatoblastoma. We preliminarily showed that knockdown of PIM1 or PIM2 did not affect hepatoblastoma cell proliferation. Further, inhibition with the pan-PIM inhibitor AZD1208 decreased tumorigenicity both *in vitro* and *in vivo*. While these data suggested that PIM3 may be the PIM kinase family member that contributes most significantly to hepatoblastoma tumorigenicity, there are currently no PIM3-specific drugs available for clinical use. More than 100 pan-PIM inhibitors have been developed with some of them currently in preclinical testing or early clinical trials, none of which specifically target PIM3. AZD1208 is one such pan-PIM inhibitor that is available for oral administration and highly selective for PIM inhibition [23, 24]. We chose this PIM inhibitor for the current investigations as it has recently been evaluated in a Phase I clinical trial in adults with advanced solid malignancies [25], potentially leading to earlier translation to pediatric administration.

The number of cell lines available for study of this rare, deadly, pediatric solid tumor is limited and imparts a significant impediment to hepatoblastoma researchers. There are currently 15 hepatoblastoma cell lines reported in the literature [26], although many lack validation and there have been instances in which some of these cell lines were determined to actually be hepatocellular carcinoma [27]. Only one of these hepatoblastoma cell lines (HuH6) is currently commercially available [26], highlighting the need for development and utilization of human hepatoblastoma PDXs as was done in the current study.

The results of AZD1208 treatment were similar between the long-term passaged cell line HuH6 and the PDX COA67, but there were some small differences. AZD1208 decreased viability of COA67 PDX cells at a low concentration of 1 μM, but there was no observed change in viability of HuH6 cells up to 20 μM. A potential reason for this finding may be the difference in derivation of the two cell lines and the environment in which they thrive. HuH6 cells are immortalized and grow rapidly in standard tissue culture whereas COA67 cells do not proliferate as readily in standard cell culture, but grow better in the *in vivo* environment. The viability studies presented were undertaken in a tissue culture environment where HuH6 cells may have been able to overcome some of the effects of the AZD1208 due to their rapid growth, whereas the viability of COA67 cells, which grow more slowly in the tissue culture environment, was more readily affected. Another potential reason for the difference in viability with PIM inhibition between the two cell lines is their underlying molecular and cellular biology. Other authors have reported that the effects of AZD1208 on viability and apoptosis vary by cancer type and cell line. In acute myeloid leukemia, AZD1208 induced apoptosis *in vitro* in some cell lines, but did not induce apoptosis at all in other cell lines [28].

A similar phenomenon was observed with the proliferation assay – 10 μM AZD1208 was required to significantly decrease proliferation in HuH6 cells, but a similar effect was seen in COA67 cells at only 1 μM. We demonstrated a decrease in Thr145 phosphorylation of p21 in both cell lines at the low 1 μM dose, indicating failure to progress through the cell cycle, despite no discernable difference in the proliferation assay. It is possible that the HuH6 cells were able to overcome the cell cycle arrest induced by p21 because their turnover rate in culture is more rapid than that of the COA67 cells and therefore, no difference was observed on the proliferation assay until higher doses of AZD1208. Additionally, p21 is a protein with complex interactions that may have seemingly both oncogenic and tumor suppressor activities depending on a multitude of factors [29].

For the *in vivo* portions of this study, AZD1208 was administered to mice bearing HuH6 subcutaneous xenografts at a dose previously demonstrated to be both well tolerated and effective in tumor reduction in a mouse model of prostate cancer [30]. Higher doses (45 mg/kg body weight/day) were noted to result in toxicity in that study. For our first animal experiment, which compared AZD1208 to vehicle control, a dose of 30 mg/kg body weight/day was given, as this dose has been used in other studies [24]. The animals’ weights were not significantly affected at this dose. The dose was decreased to 15 mg/kg body weight/day in the following experiment, which compared AZD1208 monotherapy to combination therapy with cisplatin. Even this lower dose, which is far below the doses resulting in toxicity in the previously mentioned studies, resulted in smaller tumors than cisplatin treatment alone, which is a current mainstay treatment for hepatoblastoma [18]. Additionally, the combination of cisplatin and lower dose AZD1208 decreased tumor growth even further and improved survival. While therapies for hepatoblastoma that are designed to target a single entity like PIM kinase are not likely to significantly impact outcome, using novel combinatorial therapies such as the combination of cisplatin and PIM inhibition may shift the treatment paradigm.

A strength of this study was the ability to examine both a long-term passaged cell line and a PDX. The use of PDXs is necessary in the study of hepatoblastoma given the paucity of long-term passaged cell lines and the extent to which genetic mouse models of hepatoblastoma do not reflect the human disease state and are often accompanied by hepatocellular carcinoma [26, 31]. Additionally, when long-term passaged cell lines have been studied in other conditions, results have been affected by culture conditions such as time in culture and passage number [32], making them a less than ideal model to study diseases like hepatoblastoma. With the development of PDXs, a model was introduced that more closely mimicked human disease. Few hepatoblastoma PDXs have been examined and reported in the literature. The first study describing the development of a human hepatoblastoma PDX was not published until 1996 [33]. Only a handful of papers have published research utilizing hepatoblastoma PDXs [26], providing novelty to the current study. Numerous researchers have found that tumor histomorphology and gene expression profiles of PDXs in various solid tumor types closely resemble that of the parent tumor [34–36]. The histomorphology of the COA67 PDX in the current study also closely resembled that of the parent tumor. There has been evidence and some concern regarding clonality and selection pressure with serial passaging of PDXs [37], but investigators have shown that their response to drug treatments and genetic heterogeneity is stable across multiple passages, supporting the phenotypic stability across multiple passages in mice [38–40]. For each *in vitro* experimental replicate using COA67 cells in our study, a different PDX was harvested to yield the cells. Given that the response to AZD1208 remained similar across cells from individual tumors, our data support those that have previously demonstrated that PDX phenotypes remain stable across multiple passages.

A limitation of this study is that we were unable to study the *in vivo* effects of PIM inhibition utilizing the COA67 PDX. While these tumors grow predictably when minced chunks of tumor are injected subcutaneously, it is difficult to quantify the number of cells in those injections to ensure that the same numbers of cells are injected into each mouse. When dissociated cells were injected subcutaneously, which allowed for quantification of cells, this PDX grew erratically such that very large numbers of animals would be required to overcome the standard error and demonstrate a difference between groups. While this variability in growth makes it difficult to study *in vivo*, this perceived disadvantage of PDXs may actually be an advantage for *in vitro* preclinical drug testing because their behavior is more similar to actual behavior of these tumors in humans [41].

In summary, these data demonstrated for the first time that hepatoblastoma tumorigenicity was decreased both *in vitro* and *in vivo* by PIM kinase inhibition. Another novel aspect of this study was the use of both a hepatoblastoma PDX in addition to a long-term passaged hepatoblastoma cell line. These results suggest that PIM inhibitors may be useful as a novel therapeutic for children with hepatoblastoma.

## MATERIALS AND METHODS

### Cells and cell culture

Cell lines were maintained at 37°C and 5% CO_2_. The human hepatoblastoma cell line, HuH6, was acquired from Thomas Pietschmann (Hannover, Germany) [42] and maintained in Dulbecco's Modified Eagle's Medium supplemented with 10% fetal bovine serum (HyClone, GE Healthcare Life Sciences, Logan, UT), 1 μg/mL penicillin/streptomycin (Gibco, Carlsbad, CA), and 2 mmol/L l-glutamine (Thermo Fisher Scientific, Waltham, MA). The human hepatoblastoma patient-derived xenograft (PDX), COA67, was developed as described below. Individual cells were obtained by dissociating the COA67 xenograft tumors using the Papain Dissociation System (Worthington Biochemical Corporation, Lakewood, NJ). COA67 cells were maintained in Dulbecco's Modified Eagle's Medium/Ham's F12 supplemented with 2 mmol/L l-glutamine (Thermo Fisher Scientific), 1 μg/mL penicillin/streptomycin (Gibco), 20 ng/mL epidermal growth factor (EMD Millipore, Billerica, MA), 20 ng/mL beta-fibroblast growth factor (EMD Millipore), 2% B27 supplement (Gibco), and 2.5 μg/mL amphotericin B (HyClone). Both HuH6 and COA67 cell lines were verified within the last 12 months using short tandem repeat analysis (Heflin Center for Genomic Sciences, UAB, Birmingham, AL). Real-time qPCR was performed to assess the percentage of human and mouse DNA contained in the COA67 PDX to ensure that the tumor did not contain mouse contamination (TRENDD RNA/DNA Isolation and TaqMan QPCR/Genotyping Core Facility, UAB, Birmingham, AL).

### Establishing patient-derived xenograft

The study was approved by the University of Alabama at Birmingham Institutional Review Board (X130627006) and Institutional Animal Care and Use Committee (IACUC-09803). After obtaining written informed consent, hepatoblastoma tumor tissue was obtained fresh from a primary hepatoblastoma tumor undergoing surgical excision. An additional piece of the surgical specimen was placed in formalin and embedded in paraffin for immunohistochemistry. To establish the COA67 PDX, fresh tissue was kept in RPMI 1640 medium on ice for transport and 27 mm^3^ chunks were transplanted in a sterile fashion subcutaneously in the flank of female NOD SCID mice (Envigo, Prattville, AL) under anesthesia with 3% inhalational isoflurane. Mice were maintained in pathogen-free animal housing. Tumor volumes were measured with calipers and calculated with the standard formula (width^2^ x length)/2, where the length is the largest measurement. When tumors reached 2000 mm^3^, they were harvested, chopped, and sequentially implanted from mouse to mouse to expand xenograft numbers by injecting subcutaneously into the flank of six-week-old athymic nude mice (Envigo) in 25% Matrigel™ (Corning Life Sciences, Corning, NY). Separate portions of the tumor were dissociated to be placed into cell culture as above.

### Antibodies and reagents

Rabbit monoclonal anti-PIM1 (3247), anti-PIM2 (4730), anti-PIM3 (4165), anti-BAD (9239), and anti-phospho S112 BAD (5284) were from Cell Signaling Technology (Beverly, MA). Rabbit polyclonal anti-cleaved caspase 3 (AB3623) and anti-cleaved PARP (AB3565) were from EMD Millipore. Rabbit polyclonal anti-phospho T145 p21 (ab47300) was from Abcam (Cambridge, MA). Mouse monoclonal anti-p21 (554262) was from BD Biosciences (San Jose, CA). Mouse monoclonal anti-β-actin (A1978) was from Sigma Aldrich (St. Louis, MO). AZD1208 was obtained from Selleck Chemicals (Houston, TX) and cisplatin from Sigma Aldrich.

### siRNA transfection

HuH6 cells (4 × 10^5^) were transfected for 5 days with either PIM1,2, or 3 or control small interfering RNAs (siRNAs) at 20 nM concentration with Lipofectamine^®^ RNAiMax (Thermo Fisher Scientific). PIM3 siRNA was obtained from Dharmacon (Dharmacon, GE Life Sciences, Lafayette, CO) as an ON-TARGETplus™ SMARTpool and as each of the component siRNAs individually (#8-GGCCGUCGCUGGAUCAGAU, #9-GCAGGACCUCUUCGACUUU, #10-GCGUGCUUCUCUACGAUAU, and #11-GGACGAAAAUCUGCUUGUG). PIM1 and PIM2 siRNAs were obtained from Dharmacon as ON-TARGETplus™ SMARTpools (PubChem SID 223403247 and 223403053, respectively). Control siRNA (siNeg) was obtained from Dharmacon (ON-TARGETplus™ Non-targeting siRNA #1 with the sequence UGGUUUACAUGUCGACUAA).

### Immunoblotting

Whole-cell lysates were isolated using radioimmunoprecipitation assay (RIPA) buffer supplemented with protease inhibitors (Sigma Aldrich), phosphatase inhibitors (Sigma Aldrich), and phenylmethanesulfonylfluoride (Sigma Aldrich). Lysates were centrifuged at 14 000 rpm for 1 hour at 4°C. Protein concentrations were determined using Pierce™ BCA Protein Assay reagent (Thermo Fisher Scientific) and separated by electrophoresis on sodium dodecyl sulfate polyacrylamide (SDS-PAGE) gels. Antibodies were used according to the manufacturers’ recommended conditions. Molecular weight markers (Precision Plus Protein Kaleidoscope, Bio-Rad, Hercules, CA) were used to confirm the expected size of the proteins of interest. Immunoblots were developed with Luminata Classico or Crescendo Western HRP Substrate (EMD Millipore). Blots were stripped with stripping solution (Bio-Rad) at 65°C for 20 minutes and then re-probed with selected antibodies. Equal protein loading was confirmed using β-actin.

### Cell viability, proliferation, and growth

Cell viability was measured using the alamarBlue® Cell Viability Assay (Thermo Fisher Scientific). HuH6 or COA67 cells (1.5 × 10^4^ per well) were plated in 96-well plates, allowed to rest overnight, and treated with AZD1208 at increasing concentrations for 24 hours. Following treatment, 10 μL of alamarBlue® reagent was added to each well and the absorbance was read at 562 nm (reduced reagent) and 595 nm (oxidized reagent) using a microplate reader (BioTek Gen5, BioTek, Winooski, VT). After subtracting background absorbance of the media alone, reduction of alamarBlue® reagent was calculated according to the manufacturer's protocol. Viability was reported as fold change.

Cell proliferation was measured using the CellTiter 96® Aqueous Non-Radioactive Cell Proliferation Assay (Promega, Madison, WI). HuH6 or COA67 cells (5 × 10^3^ per well) were plated in 96-well plates, allowed to rest overnight, and treated with increasing concentrations of AZD1208 for 24 hours. Following treatment, 10 μL of CellTiter 96® reagent was added to each well and the absorbance was read at 490 nm using a microplate reader (BioTek Gen5) to detect the formazan product. The background absorbance of the media alone was subtracted and proliferation was reported as fold change.

### Migration and invasion

For migration assays, 24-well culture plates (Corning Life Sciences) were utilized. The bottom of the 8 μm micropore Transwell® inserts were coated with collagen I (10 μg/mL, MP Biomedicals, Santa Ana, CA) for HuH6 studies or fibronectin (10 μg/mL, Qiagen, Germantown, MD) for COA67 studies overnight at 37°C and then washed with phosphate-buffered saline (PBS). The inserts were placed in wells containing 333 μL media with the appropriate concentration of AZD1208 and treated for 24 hours. Treated cells (3 × 10^4^ HuH6 or 3 × 10^5^ COA67) were placed inside each insert and allowed to migrate for 24 or 72 hours (HuH6 and COA67, respectively). The inserts were fixed with 3% paraformaldehyde and stained with 1% crystal violet. Images of the inserts were obtained using a light microscope and the number of cells were counted using ImageJ (https://imagej.nih.gov/ij). Migration was reported as fold change in number of cells migrating through the membrane.

Similar methods were used for invasion. The inside of the 8 μm micropore Transwell® inserts were coated with Matrigel™ (1 mg/mL, 50 μL; BD Biosciences) overnight at 37°C and then washed with PBS. For COA67 studies, the bottom of the inserts were also coated with fibronectin (10 μg/mL, Qiagen) overnight at 37°C and then washed with PBS. The inserts were placed in wells containing 333 μL media with the appropriate concentration of AZD1208. Cells were treated with AZD1208 for 24 hours, after which 3 × 10^4^ (HuH6) or 3 × 10^5^ (COA67) cells were placed inside each insert and allowed to invade for 24 or 72 hours (HuH6 and COA67, respectively). Fixation, staining, image acquisition, and counting was performed as described above. Invasion was reported as fold change in number of cells invading through the Matrigel™ and membrane.

Cell migration was also evaluated utilizing a monolayer wound-healing assay. A standard scratch was made in the well with a sterile 200 μL pipette tip. Images were obtained of the scratch wound at 0, 24, 48, and 72 hours. The area of the wound in pixels was quantified using the ImageJ MRI Wound Healing Tool. Data were reported as fold change scratch area and compared between groups.

### Attachment-independent growth

Attachment-independent growth was determined using the soft agar assay. A layer of 2× culture media and 1% noble agar (BD Biosciences) in a 1:1 ratio was poured into 60 × 15 mm petri dishes and allowed to cool. A second layer containing the same ratio of culture media and agar, but also HuH6 cells (1 × 10^4^ per dish), was added. Dishes were treated with AZD1208 in fresh media twice weekly. After colonies were visible by eye, the dishes were imaged using Gel Dock Imager (Bio-Rad) and Quantity One software (Bio-Rad). The number of colonies per dish were counted using ImageJ.

### Cell cycle analysis

HuH6 cells (1 × 10^6^) were plated, allowed to attach overnight, and then treated with AZD1208 (0, 10, 20 μM) for 72 hours. Cells were lifted with trypsin, washed with PBS, and fixed in cold 100% ethanol for at least 30 minutes. Cells were then washed in PBS and stained for 30 minutes at room temperature with a solution containing 20 μg/mL propidium iodide (Invitrogen, Carlsbad, CA) and RNAse A (0.2 mg/mL; Invitrogen) in 0.1% Triton X (Active Motif, Carlsbad, CA). Data for flow cytometric analysis of the cell cycle were obtained using a FACSCalibur flow cytometer (Becton Dickinson Biosciences) and analyzed with FlowJo software (Becton Dickinson).

### *In vivo* studies

Six-week old female athymic nude mice (Envigo) were maintained in the specific pathogen-free facility with standard 12-hour light/dark cycles and access to chow and water *ad libitum*. Experiments were approved by the Institutional Animal Care and Use Committee (IACUC-9803) and conducted within institutional, national, and NIH guidelines. Tumor volumes were measured with calipers and calculated with the standard formula (width^2^ x length)/2, where the length is the largest measurement.

HuH6 human hepatoblastoma cells (2.5 × 10^6^ in 25% Matrigel™; BD Biosciences) were injected subcutaneously into the right flank of 30 mice. When tumor size reached an average of 150 mm^3^, mice were randomized to receive vehicle (ORA-Plus®, 50 μL; Perrigo, Allegan, MI) or AZD1208 (30 mg/kg body weight/day in ORA-Plus®, 50 μL) by oral gavage for a total of 14 days (n = 15 per group). Animals were humanely euthanized with CO_2_ and cervical dislocation 4 hours after the last vehicle or drug administration, and the tumors were harvested and prepared for further study.

For the cisplatin/AZD1208 combination study, HuH6 cells (2.5 × 10^6^ in 25% Matrigel™; BD Biosciences) were injected subcutaneously into the right flank of 12 mice. When tumor size reached greater than 45 mm^3^, mice were randomized to receive cisplatin alone (2 mg/kg body weight/day; Sigma Aldrich), AZD1208 alone (15 mg/kg body weight/day; Selleck Chemicals), or combination treatment with cisplatin and AZD1208 administered in the same doses as in the single agent groups (n = 4 per group). Cisplatin (or sterile saline for AZD1208 alone group) was administered by intraperitoneal injection on days 1-3 and 14-16. On the remaining days (days 4-13 and 17-30), AZD1208 in ORA-Plus® (or ORA-Plus® alone for cisplatin alone group) was administered by oral gavage in a total volume of 50 μL. Animals were treated for 30 days and followed after the last day of treatment for survival. When IACUC parameters were reached, the animals were humanely euthanized and the tumors harvested.

### Immunohistochemistry

Formalin-fixed paraffin-embedded human or xenograft tumor specimens were sectioned into 6 μm sections and baked at 70°C for one hour on positive slides. Slides were deparaffinized, steamed, quenched with 3% hydrogen peroxide, and blocked with blocking buffer (BSA, powdered milk, Triton X-100, PBS) for 30 minutes at 4°C. The primary antibodies anti-Ki67 (rabbit polyclonal, 1:600, ab11580, Abcam), anti-PIM3 (rabbit polyclonal, 1:500, ab71321, Abcam), and anti-phospho S112 BAD (rabbit monoclonal, 1:50, #5284S, Cell Signaling Technology) were added and incubated for 1 hour in a humidity chamber at room temperature. After washing with PBS, the secondary antibodies for mouse or rabbit (R.T.U. biotinylated universal antibody, Vector Laboratories, Burlingame, CA) were added for 1 hour at 22°C. The staining reaction was developed with VECTASTAIN Elite ABC reagent (PK-7100, Vector Laboratories) and Metal Enhanced DAB Substrate (Thermo Fisher Scientific). Slides were counterstained with hematoxylin. Negative controls (mouse IgG, 1 μg/mL, Invitrogen, or rabbit IgG, 1 μg/mL, EMD Millipore) were included with each run.

Stained slides were evaluated by two pathologists (S.M., E.M.M.) blinded to the patients and treatment groups. For Ki67 staining, the number of Ki67 cells per 500 cells in a representative section of each tumor was counted and the mean was calculated and reported [43]. For phospho-BAD staining, slides were scored based upon the intensity of the stain and the percentage of tumor cells staining positive. A stain score was calculated as the product between the degree of staining (no stain = 0, weak = 1, moderate = 2, strong = 3) and percentage of positive cells, such that specimen with strong staining in 50 percent of the cells would have a stain score of 150 (3 × 50 = 150).

### Data analysis

Densitometry of immunoblots was performed utilizing Scion Image Program (http://www.nist.gov/lispix/imlab/prelim/dnld.html). Each band was normalized to background. PIM bands were normalized to their respective β-actin, and all bands were then normalized to siNeg group as previously reported [44]. Experiments were repeated at least in triplicate and data were reported as mean ± standard error of the mean (SEM). Student's *t*-test or ANOVA was used as appropriate, with p ≤ 0.05 determined to be statistically significant. Kaplan-Meier with log rank analysis was performed for animal survival studies.

## SUPPLEMENTARY MATERIALS FIGURES



## Abbreviations

PIM kinase: Proviral Integration site for Maloney murine leukemia virus kinase
PDX: Patient derived xenograft
