# Supplementary material for: Targeting PIM kinase as a therapeutic strategy in human hepatoblastoma
Source: Oncotarget. 2018 Apr 27;9(32):22665–79. doi: 10.18632/oncotarget.25205 (PMC5978256; doi:10.18632/oncotarget.25205)
Supplement: Supplementary file 1 [file oncotarget-09-22665-s001.pdf]

Targeting PIM kinase as a therapeutic strategy in human hepatoblastoma

SUPPLEMENTARY MATERIALS

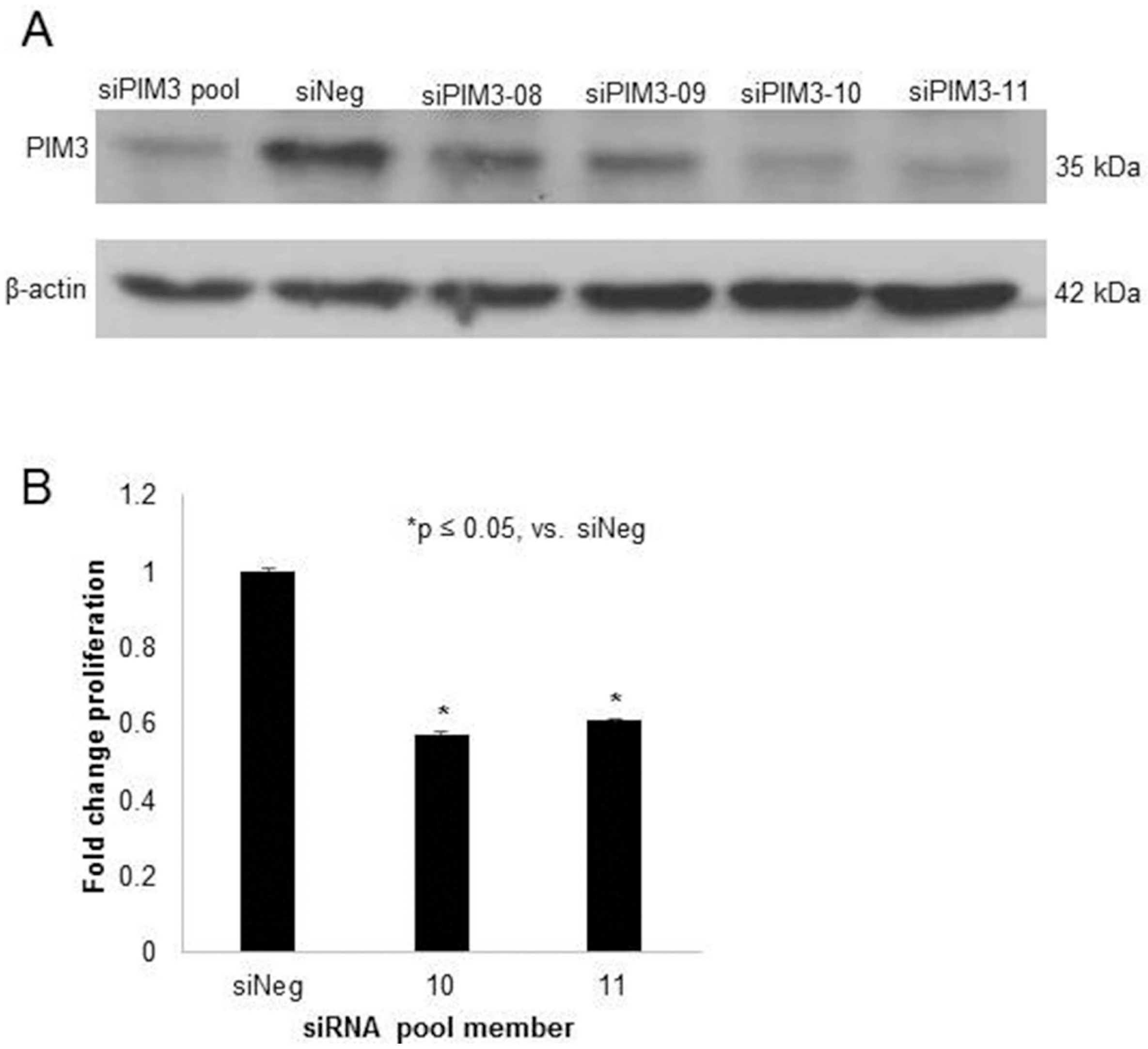

Supplementary Figure 1: Inhibition of PIM3 with siRNA. (A) Immunoblotting for PIM3 in lysates from HuH6 cells transfected with siPIM3 pool, control siRNA (siNeg), or the four members of the SMARTpool (siPIM3-08, siPIM3-09, siPIM3-10, and siPIM3-11) was performed. All members of the siPIM3 pool decreased PIM3 expression, with siPIM3-10 and siPIM3-11 achieving the most knockdown of PIM3. **(B)** Following 5 days of transfection with siPIM3-10, siPIM3-11, or siNeg, proliferation was assessed. HuH6 cells transfected with siPIM3-10 and siPIM3-11 had significantly decreased proliferation compared to HuH6 cells transfected with the siNeg control, experiments were repeated at least three times and data reported as fold change ± SEM.

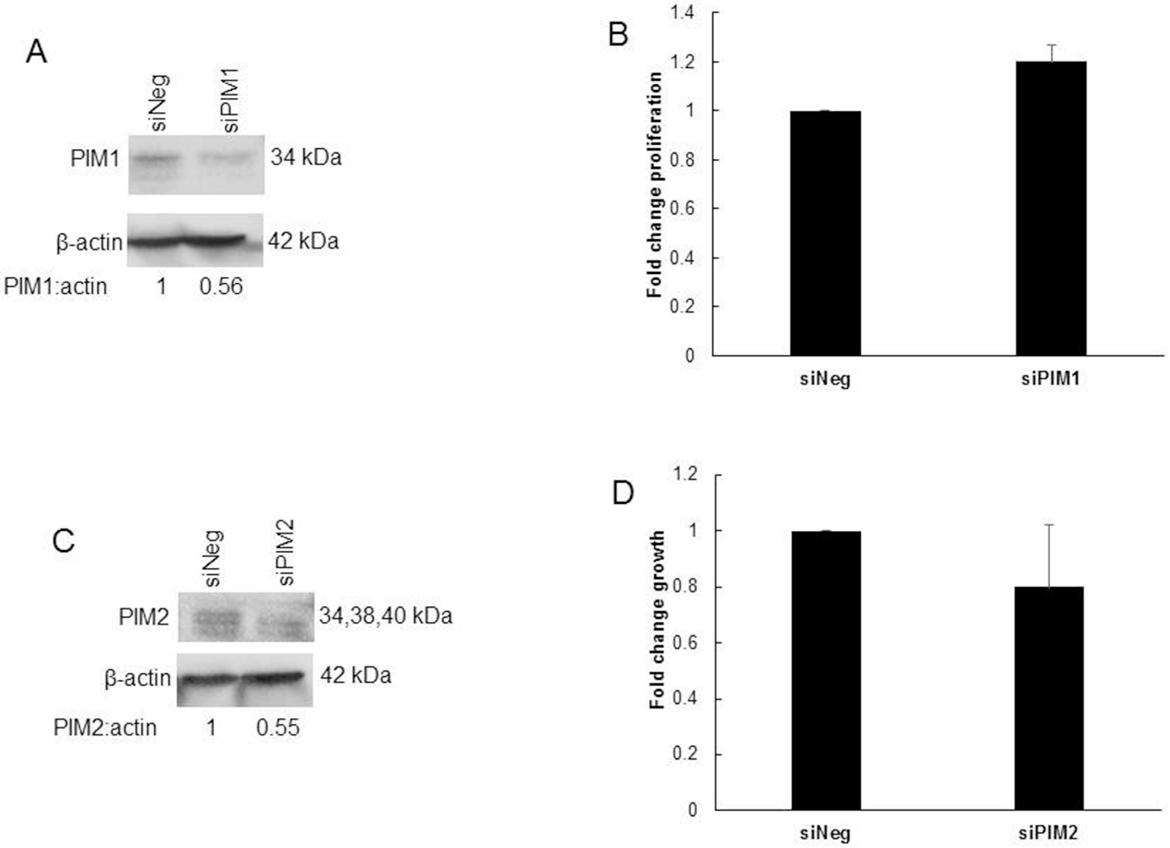

Supplementary Figure 2: Inhibition of PIM1 and PIM2 with siRNA. (A) Immunoblotting for PIM1 in HuH6 cells transfected with control siRNA (siNeg) or siRNA targeting PIM1 (siPIM1) revealed a decrease in expression of PIM1 in the siPIM1 cells. Band density was verified with densitometry. **(B)** Following 5 days of transfection with siNeg or siPIM1, proliferation was assessed. There was no significant difference between siPIM1 and siNeg cells. **(C)** Immunoblotting for PIM2 in HuH6 cells transfected with control siRNA (siNeg) or siRNA targeting PIM2 (siPIM2) revealed a decrease in expression of PIM2 in the siPIM2 cells. Band density was verified with densitometry. **(D)** Following 5 days of transfection with siNeg or siPIM2, growth was assessed by manually counting the number of live cells, as assessed by exclusion of trypan blue, after 48 hours in culture. There was no significant difference in cell growth between siPIM2 and siNeg cells.

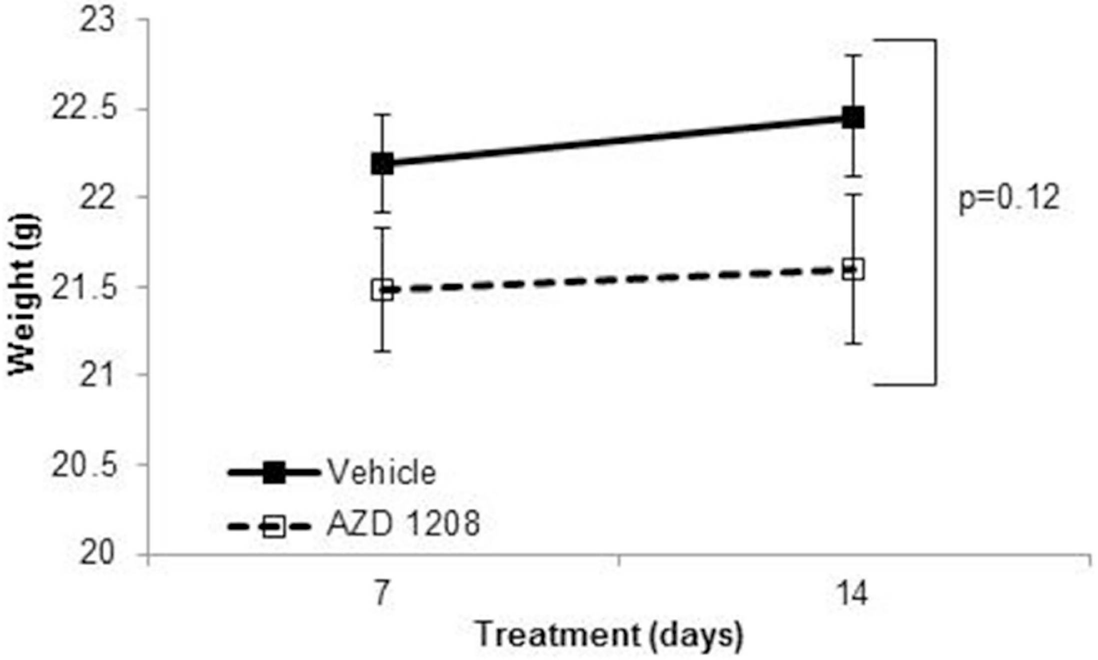

Supplementary Figure 3: AZD1208 treatment did not significantly affect animal weight. Animals with HuH6 flank tumors received either AZD1208 (30 mg/kg body weight/day in ORA-Plus®, 50 μL) or vehicle alone (ORA-Plus®, 50 μL) by oral gavage for a total of 14 days (n = 15 per group). They were weighed following one week of treatment and at the time of euthanasia (14 days). There was no significant difference in the weight of the animals in the two groups, indicating that AZD1208 did not significantly affect mouse growth.

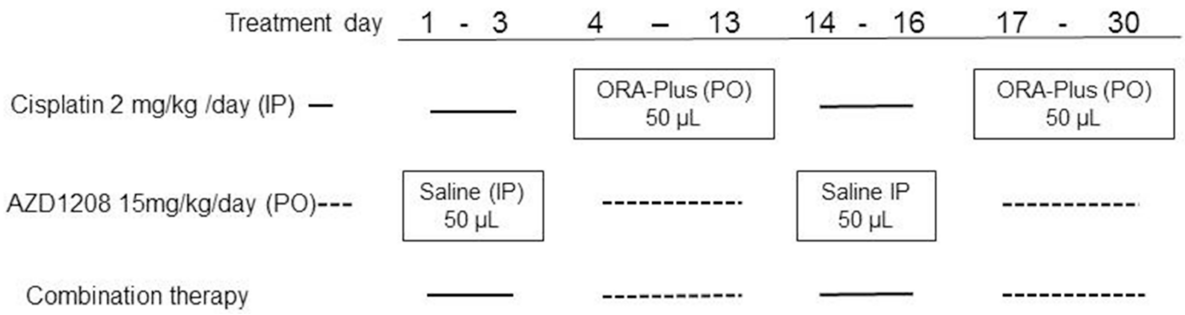

Supplementary Figure 4: *In vivo* treatment scheme for combination AZD1208 and cisplatin. Cisplatin, indicated by the solid line, was administered by intraperitoneal injection on days 1-3 and 14-16 to mice in the cisplatin alone and combination therapy groups. For the AZD1208 alone group, mice received sterile saline by intraperitoneal injection on days 1-3 and 14-16. On days 4-13 and 17-30, AZD1208 in ORA-Plus®, indicated by the dashed line, was administered by oral gavage in a total volume of 50 µL to the AZD1208 alone and combination therapy groups. For the cisplatin alone group, mice received ORA-Plus® by oral gavage on days 4-13 and 17-30.

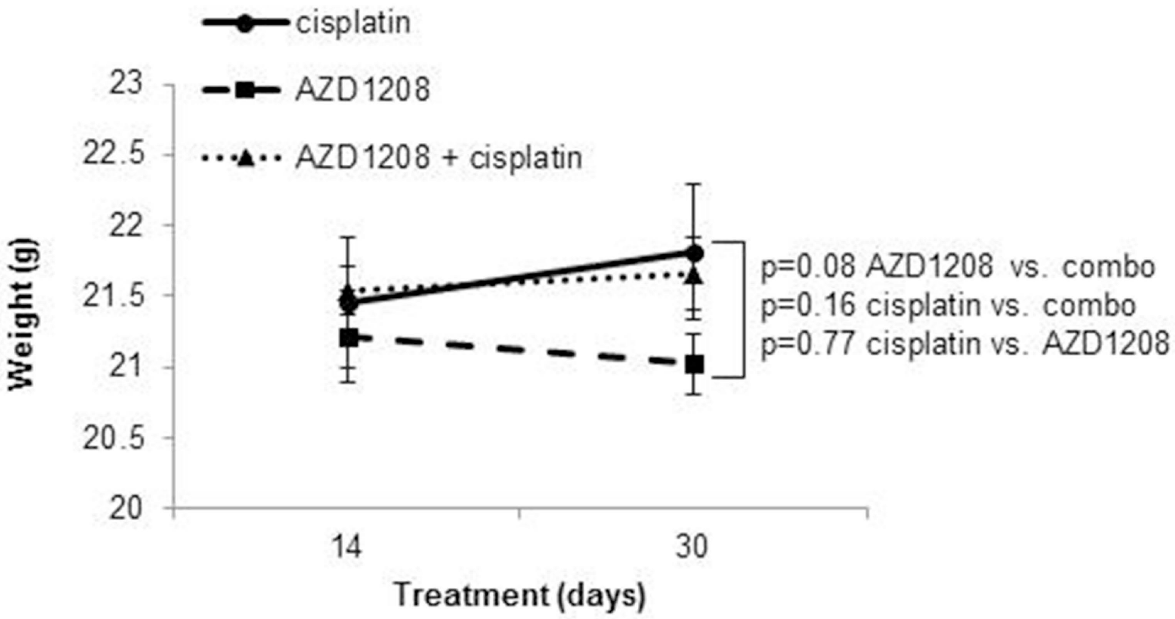

Supplementary Figure 5: Combination treatment with cisplatin and AZD1208 did not significantly affect animal weight. Animals with HuH6 flank tumors received either cisplatin alone, AZD1208 alone, or combination treatment with cisplatin and AZD1208 administered in the same doses as the single agent groups. Animals were treated for 30 days and their weights followed over time. There was no significant difference in the weight of the animals in the three groups, indicating that AZD1208 combined with cisplatin did not significantly affect mouse growth.
